# Association among serum uric acid, hyperactivity, impulsivity and dietary components in adults: a cross-sectional study Uric acid, hyperactivity/impulsivity symptoms, and dietary components in adults

**DOI:** 10.1371/journal.pone.0343566

**Published:** 2026-03-02

**Authors:** Roberto A. Molina-Campuzano, Mariela Bernabe-Garcia, Felipe Vazquez-Estupiñan, Leonel Jaramillo-Villanueva, Maricela Rodriguez-Cruz, Ricardo Saracco-Alvarez, Claudia Sanchez-Sanchez, Israel Moreno-Moreno, Irma S. Y. Corlay-Noriega

**Affiliations:** 1 Unidad Médica de Consulta de Atención Psiquiátrica, Hospital Psiquiátrico “Morelos”. Instituto Mexicano del Seguro Social. México City, Ciudad de México, México; 2 Medical Research Unit in Nutrition, UMAE Hospital de Pediatría, Instituto Mexicano del Seguro Social. Mexico City, Ciudad de México, México; 3 Psychiatric Service, Hospital Angeles Clínica Londres. México City, Ciudad de México, México; 4 Department of Mental Health, UMAE Hospital de Pediatría, Instituto Mexicano del Seguro Social. México City, Ciudad de México, México; 5 Biomedical Research in Mental Health. Instituto Nacional de Psiquiatría, Ramón de la Fuente Muñiz. México City, Ciudad de México, México,; 6 Psychiatric Service, UMAE Hospital de Especialidades, Instituto Mexicano del Seguro Social. México City, Ciudad de México, México; BRAC University, BANGLADESH

## Abstract

**Background:**

A positive association has been reported between elevated serum levels of uric acid (UA) with impulsivity and hyperactivity, behavior in murine models, and human psychopathologies. Still, other factors, such as diet composition, have not been considered. This study aims to determine the association between serum UA, impulsivity/hyperactivity symptoms, and dietary components in adults with psychiatric disorders.

**Methods:**

A prospective cross-sectional study was conducted on 128 adults who attended a psychiatric service. Fasting serum UA levels were determined by spectrophotometry. Impulsivity and hyperactivity symptoms were evaluated using the Adult ADHD Self-Report Scale (ASRS). Dietary components, including macronutrients, fiber, fructose, added sugar, vitamin C, zinc, copper, n-3 fatty acids, caffeine, and alcohol, were estimated using a 24-hour recall, food models, and nutrition software. Spearman's correlation and general linear models were applied. The last was used to adjust for confounders.

**Results:**

Serum UA levels were positively correlated to symptoms of hyperactivity/impulsivity and hyperactivity (*Rho* = 0.206, *p*= 0.020; *Rho* = 0.194, *p*= 0.028, respectively). Those correlations remained significant after adjusting for confounders. Every 1 mg/dl increase in serum UA levels predicted an elevation of 1.5 points of the hyperactivity/impulsivity symptoms (*p*= 0.002; *p-*model< 0.001) and 1 point of the hyperactivity symptoms (*p*= 0.003; *p-*model < 0.001). Hyperactivity/impulsivity symptoms together and separated were positively correlated with depression (*Rho* = 0.470, *Rho* = 0.389, *Rho* = 0.485; all *p*< 0.001, respectively). Serum UA levels negatively correlated with dietary intake of total fiber, vitamin C, and copper but positively with waist circumference (*Rho* = −0.297, *Rho* = −0.185, *Rho* = −0.212, and *Rho* = 0.203, all *p <* 0.05, respectively). Hyperactivity correlated with dietary zinc, while impulsivity correlated with alcohol consumption (*Rho* = −0.185 and *Rho* = 0.195, *p*< 0.05, respectively).

**Conclusion:**

The serum UA concentration independently predicts hyperactivity/impulsivity symptoms in adults with psychiatric disorders.

## 1. Introduction

Uric acid (UA) is a product of both exogenous and endogenous purine nucleotide metabolism. The exogenous pool is highly variable due to dietary or environmental factors, whereas endogenous UA production results from the metabolism of the liver and other tissues. UA is excreted by the intestines and kidneys [1].

The UA is also a product of the purinergic system, which is involved in neurodevelopment and pathophysiological processes, such as cell proliferation and differentiation, neuronal-glial interaction, and inflammation. The amount of UA is inversely proportional to the amount of adenosine at the extracellular level of the neuron, and it acts through two adenosine receptors, A1R and A2AR. A typical concentration of adenosine has been observed to result in a decrease in motor activity. Conversely, an increase in UA has been linked to a rise in either impulsivity or hyperactivity [2].

Elevated serum UA concentrations in humans are notably elevated in Lesch-Nyhan syndrome, characterized by impulsive behavior [3]. In addition, high serum UA concentrations have been associated with several maladaptive traits that often overlap among several psychiatric disorders [4], including thrill-seeking [5], hyperactivity, impulsivity, irritability [6], and attention-deficit/hyperactivity (ADHD) [7]. ADHD is a common neurodevelopmental disorder characterized by hyperactivity, impulsivity, and inattention that impair functioning and contribute to the difficulties experienced in their daily lives, affecting school-age children [8] with 55.3% persistence in adulthood [9]. In childhood, girls often present inattentive symptoms rather than hyperactive/impulsive or combined ADHD compared to boys, which may go unnoticed and misdiagnosed. These results in a 1.5–2.5 times higher rate of ADHD in boys relative to girls, but become more similar between them by adulthood [10].

Endogenous UA can also be modified by genetic factors. Studies conducted to estimate the heritability of serum urate levels have indicated that genetic factors account for 25% to 60% of the variability in individuals of European ancestry. Therefore, nongenetic factors such as environmental exposures and diet explain the remaining variability. The consumption of red meat, seafood, sugary beverages, and alcoholic beverages has been associated with increased serum UA levels [11]. Meat and seafood are good sources of protein and purines [12], while sugary beverages are sources of added sugar and fructose [13]. Alcohol intake was also associated with impulsivity [14]. In contrast, fiber, vitamin C, and some minerals reduced serum UA concentrations [15]. Moreover, dietary components such as macronutrients, vitamins, and minerals from diet and supplements have been associated with ADHD in young adults [16] and children [17].

The presence of hyperuricemia also increased the stimulation of the cerebral cortex, likely due to its structural similarity to caffeine, a central nervous system stimulant [18]. Caffeine, on the other hand, was associated with reduced serum UA concentrations [13]. Other unhealthy style behaviors and metabolic consequences like cigarette smoking, physical inactivity, obesity, and metabolic syndrome have also been related to impulsivity and elevated serum UA concentrations [14,19–22].

While some reports suggest a connection between serum UA concentration, behavior, and diet composition, the relationship among these factors has not been thoroughly examined together. A more comprehensive understanding of the predictive factors and symptoms associated with hyperactivity and impulsivity may result in more effective management strategies.

This study aims to determine the association among levels of serum UA, impulsivity, hyperactivity symptoms, and dietary factors in adults with psychiatric disorders.

## 2. Materials and methods

### 2.1. Study design and participants

**Fig 1:**
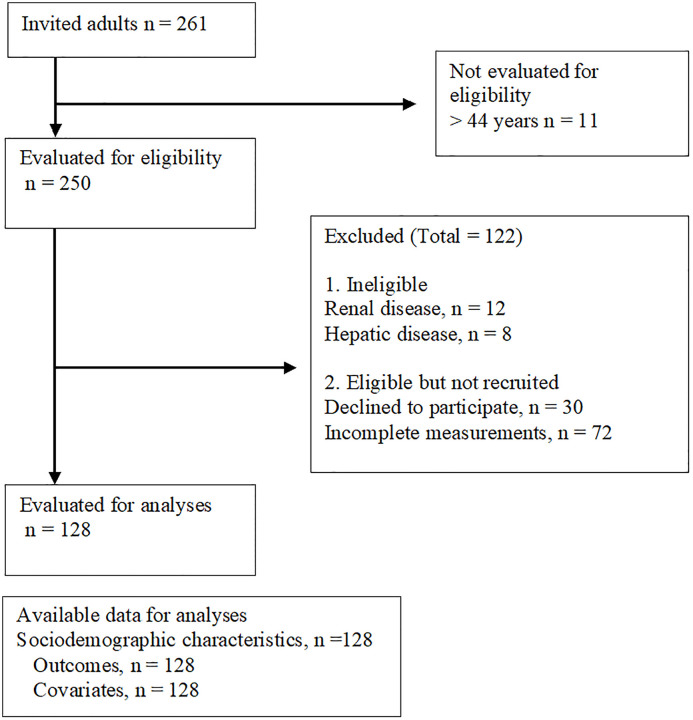
STROBE flow diagram.

A cross-sectional study was conducted on 128 subjects following STROBE guidelines (Fig 1) [23] in a third-level hospital of the Instituto Mexicano del Seguro Social (IMSS) in Mexico City, Mexico. The research project adhered to the principles outlined in the Belmont Report and the Declaration of Helsinki regarding medical research involving humans and was approved by the Local Ethics Committee of the IMSS (No. 2021-3601-222). Written informed consent was obtained from all participants before the commencement of the study, along with the signatures of two witnesses.

Data privacy. All research information, including printed and electronic files, as well as written consents, was kept confidential and securely stored under lock and key. Access to information was exclusively granted to the corresponding author, who provided access only to research staff under strict supervision to ensure the protection of participant information during its capture in the database.

Participants who attended the psychiatric service from March 1st, 2022, to August 30th, 2022, were eligible. Selection criteria were age between 18 and 44 years [24], with a mental disorder according to the 10th revision of the International Classification of Diseases (ICD-10), and clinical interviews performed by a psychiatrist confirmed the diagnoses. The subjects with the following diagnoses were excluded: Lesch-Nyhan [3], Seegmiller syndrome, Hers disease, Von Gierke disease [25], psoriasis, and hepatic or renal disease [26]. Subjects who were receiving treatment with cyclosporine, ethambutol [27], pyrazinamide, losartan, benzbromarone, probenecid, sulfinpyrazone, and xanthine oxidase inhibitors [27,28] retrieved from electronic records, and those taking dietary supplements were excluded.

### 2.2. Measures

#### 2.2.1. Demographic and clinical information.

Demographic factors included were psychiatric disorders, biological sex, age in years, and academic education stratified by elementary school, high school, professional school, or postgraduate.

#### 2.2.2. Impulsivity and hyperactivity assessment.

All subjects completed the Adult ADHD Self-Report Scale (ASRS), an 18-item self-report questionnaire that assesses inattention and hyperactivity/impulsivity over the last six months. The hyperactivity/impulsivity section of the ASRS scale was used as a dimensional symptom measure of hyperactivity and impulsivity, not as a diagnostic tool, analyzing them together (as hyperactivity/impulsivity symptoms) and separately (as hyperactivity or impulsivity symptoms) [29,30]. A higher score indicates a greater number of symptoms.

#### 2.2.3. Measurement of uric acid.

All subjects underwent venipuncture between 6:00 a.m. and 7:00 a.m. after an overnight fast of 12 hours. The blood samples were analyzed on the same day as the venipuncture. Serum concentrations of UA (mg/dl) were measured using spectrophotometry with commercial kits and their internal controls (Beckman Coulter, Brea, California, USA) and an automatic biochemical analyzer (Sysmex XN-9000, Roche, Basel, Switzerland).

#### 2.2.4. Measurement of dietary factors.

Food intake was assessed through a 24-hour recall conducted by a nutritionist, which included two non-consecutive weekdays and 1 weekend day to estimate daily energy and nutrient intake [31]. The portion size consumed was calculated by presenting food models and various cup and spoon sizes to obtain the grams or milliliters of each food, and then analyzed using Food Processor Nutrition Analysis Software 11.6 v. Esha Research. Dietary protein, carbohydrate, fat, fructose, added sugar, total fiber intake, vitamin C, copper, zinc, and omega-3 fatty acids intake were estimated in g/1000 kcal; caffeine was estimated in mg/day [15,32,33]. Alcohol consumption was based on the standard drink units (SDU) per month, considering one SDU = 10 g of alcohol [34].

#### 2.2.5. Assessment of covariates.

The age was recorded in years. Physical activity was measured using the International Physical Activity Questionnaire (IPAQ) and expressed as the total Metabolic Equivalent of Task (MET) minutes per week [35]. Depression symptoms were evaluated using the 21-item Beck Depression Inventory-II [36]. Smoking was measured as the monthly number of cigarettes smoked [37].

Subjects were instructed to refrain from eating or drinking for two hours before measurement. After a ten-minute standing break, height and waist circumference were assessed three times in the same position using a wall-fixed stadiometer and a metallic measurement tape, respectively, in centimeters. Then, weight and body composition were determined per duplicate with an InBody 970 (In Body Co., Ltd., Seoul, Korea) [38]. Central obesity was quantified using waist circumference [39], while obesity was defined as a percentage of total body fat ≥ 32% in women and ≥ 25% in men [40].

The biological sex (because males present higher serum UA concentrations than females) [28] and the use of psychotropic drugs (absence, presence) were reviewed in clinical records and confirmed during the interview. Finally, the metabolic syndrome was also determined, characterized by elevated waist circumference, triglycerides, blood pressure, and fasting glucose but reduced HDL cholesterol [41].

#### 2.2.6. Sample Size.

A *priori* sample size was computed to identify a correlation *r* ≥ 0.36 between UA and hyperactivity (assessed with David's Scale) [6]; the same correlation value was considered for impulsivity estimation. The values for alpha = 0.05 and beta = 0.20, with a power of 0.80, resulted in 58 subjects. Recruitment continued until a total of 128 subjects were reached, with no missing data. This sample size was selected to account for confounding factors in the multivariate general linear models.

#### 2.2.7. Statistical analysis.

The significance level was set at *p* < 0.05. Statistical analyses were performed using SPSS, version 25 software. Categorical variables are presented as frequencies and percentages. Data were grouped by biological sex because males have higher serum UA levels. Only patients with complete data were included in the analysis; thus, there was no missing data. The normality of the data distribution was evaluated using the Kolmogorov-Smirnov test for more than 50 observations. If the number of observations was 50 or lower, the Shapiro-Wilk test was applied. The median and interquartile range (IQR) were reported for descriptive statistics. Obesity was analyzed dichotomously and quantitatively in terms of percentage of body fat mass. The dietary factors were expressed as nutrient density, adjusted for 1000 kcal of dietary intake [42].

According to the data distribution, the Chi-Square test was used to compare categorical variables, while the Wilcoxon-Mann-Whitney test was used to compare quantitative variables.

Correlations were calculated using Spearman's correlation tests. Covariates were established *a priori* since the registered protocol; next, they were identified as potentially significant if *p* ≤ 0.200 resulted from group comparisons or correlations. A correlation matrix was used to determine pairwise correlations between predictive factors that suggest potential multicollinearity; those predictors were removed before constructing the model. Finally, the selected predictive factors were based on biological plausibility reported in scientific literature. General linear models were used to assess the effect of serum UA on impulsivity and hyperactivity symptoms together and separately. Main effects and a square sum type III with a simple sampling simulation of 1,000 samples were used. The assumptions for the General Linear Models were evaluated and met.

## 3. Results

No differences in sociodemographic characteristics or primary diagnosis were observed among the subjects, as shown in Table 1. The mean serum UA concentrations for the 128 adults was 4.92 ± 1.33 mg/dl, with a median hyperactivity/impulsivity score of 18.0 [IQR 12–25] points, a median hyperactivity score of 13.0 [9–18] points, and a median impulsivity score of 6.0 [3–8] points.

**Table 1:** Sociodemographic characteristics of adults.

Variables	*Women* n= 85 (66%)	*Men* n = 43 (34%)	*p*-value
Age (years)	32.0 [27.0, 37.5]	29.0 [23.0, 34.0]	0.050
*Academic education*			
Elementary school	1 (1)	0	
High school	28 (33)	21 (49)	
Professional school	49 (58)	20 (46)	
Postgraduate	7 (8)	2 (5)	0.350
*Psychiatric disorder*			
Major depressive disorder	48 (56)	20 (47)	
Generalized anxiety disorder	21 (25)	15 (35)	
ADHD	6 (7)	5 (12)	
Borderline personality disorder	6 (7)	1 (2)	
Alcohol dependence syndrome	3 (4)	1 (2)	
Obsessive-compulsive disorder	1 (1)	0	
Schizophrenia	0	1 (2)	0.224

Data are presented as frequencies (n), percentages (%), or median and interquartile range [IQR]. ADHD: Attention Deficit/Hyperactivity Disorder.

**Fig 2:**
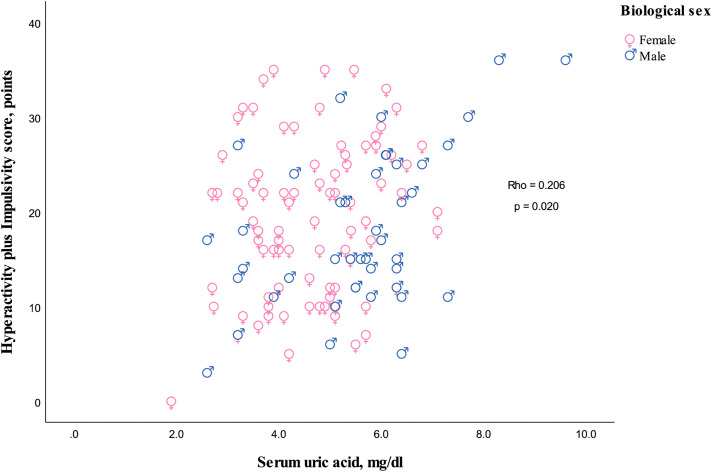
Scatterplot depicting the relationship between serum uric acid levels and hyperactivity plus impulsivity symptoms. Rho indicates Spearman’s correlation coefficient for both biological sexes, and the p-value represents the two-sided significance.

**Fig 3:**
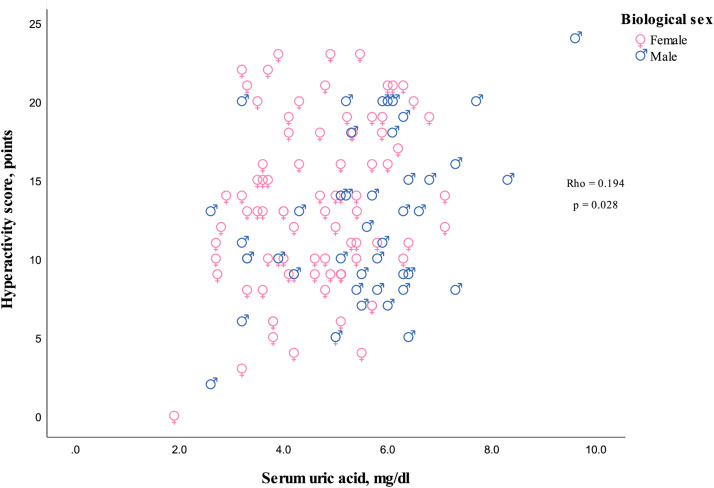
Scatterplot depicting the relationship between serum uric acid levels and hyperactivity symptoms. Rho indicates Spearman’s correlation coefficient for both biological sexes, and the p-value represents the two-sided significance.

**Fig 4:**
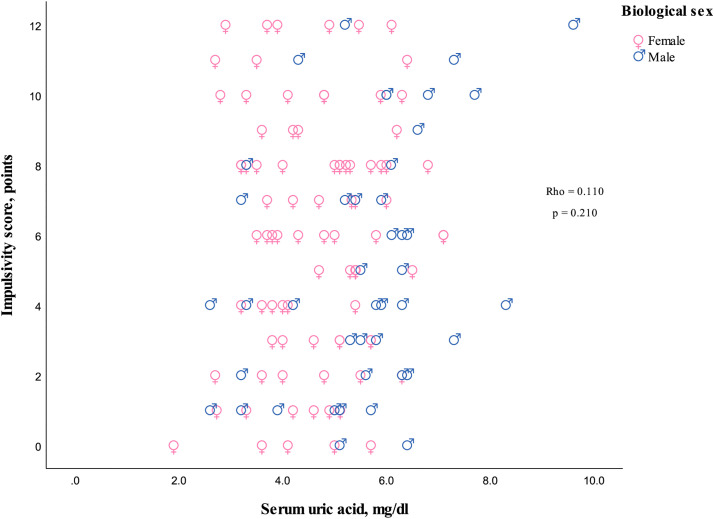
Scatterplot depicting the relationship between serum uric acid levels and impulsivity symptoms. Rho indicates Spearman’s correlation coefficient for both biological sexes, and the p-value represents the two-sided significance.

The serum UA concentrations showed a positive correlation with hyperactivity/impulsivity symptoms and separated hyperactivity symptoms, but not with impulsivity symptoms when considered separately (Figs 2–4).

Regarding correlations with dietary components, dietary zinc showed an inverse correlation with hyperactivity symptoms, although a statistical trend was observed with symptoms of hyperactivity/impulsivity and impulsivity. Serum UA concentrations negatively correlated with dietary fructose, total fiber, vitamin C, and copper. However, the correlations with other nutrients were not statistically significant (see Table 2).

**Table 2:** Correlations of hyperactivity, impulsivity symptoms, serum uric acid levels, and dietary components.

Intake of dietary components		Hyperactivity plus Impulsivity score (points)	Hyperactivity score (points)	Impulsivity score (points)	Serum uric acid (mg/dl)
Total carbohydrate (g/1000 kcal)	Coeff.	−0.034	−0.024	−0.053	0.010
	*p*-value	0.701	0.787	0.555	0.909
Total fat (g/1000 kcal)	Coeff.	0.061	0.099	0.010	−0.024
	*p*-value	0.492	0.264	0.909	0.790
Total protein (g/1000 kcal)	Coeff.	−0.086	−0.125	−0.045	−0.066
	*p*-value	0.334	0.159	0.617	0.462
Fructose (g/1000 kcal)	Coeff.	−0.012	−0.002	0.005	−0.217^*^
	*p*-value	0.892	0.978	0.957	**0.014**
Added sugar (g/1000 kcal)	Coeff.	−0.023	−0.028	−0.025	−0.023
	*p*-value	0.796	0.752	0.780	0.794
Vitamin C (mg/1000 kcal)	Coeff.	0.049	0.042	0.027	−0.185^*^
	*p*-value	0.586	0.641	0.765	**0.037**
Total fiber (g/1000 kcal)	Coeff.	−0.113	−0.129	−0.017	−0.297^**^
	*p*-value	0.205	0.147	0.851	**0.001**
Cooper (mg/1000 kcal)	Coeff.	−0.038	−0.027	−0.054	−0.212^*^
	*p*-value	0.667	0.760	0.544	**0.016**
Zinc (mg/1000 kcal)	Coeff.	−0.168	−0.185^*^	−0.150	−0.116
	*p*-value	0.057	**0.037**	0.092	0.191
Omega-3 Fatty Acids (g/1000 kcal)	Coeff.	0.067	0.078	0.056	0.142
	p-value	0.452	0.383	0.528	0.110

n = 128 subjects; Coeff.: Spearman’s Rho coefficient; mg/dl: milligrams per deciliter; g/1000 kcal: Grams per 1000 kilocalories; Sig.: * Significance < 0.05 level (Two-sided); ** Significance < 0.01 level (Two-sided).

On the other hand, depression symptoms showed a positive correlation with hyperactivity/impulsivity symptoms, together and separated, while alcohol consumption exhibited a positive correlation only with impulsivity. Finally, waist circumference positively correlated with serum UA levels. There were no correlations with other subject characteristics or behavior (see Table 3).

**Table 3:** Correlations of hyperactivity, impulsivity symptoms, serum uric acid, dietary components, and covariates.

Variables	Hyperactivity plus Impulsivity score (points)	Hyperactivity score (points)		Impulsivity score (points)	Serum uric acid (mg/dl)
Caffeine intake (mg/day)	Coeff.	−0.028	0.013	−0.025	−0.073
	*p*-value	0.751	0.881	0.782	0.412
Alcohol consumption (Units per month)	Coeff.	0.145	0.088	0.195^*^	−0.025
	*p*-value	0.102	0.325	**0.027**	0.783
Age (years)	Coeff.	0.135	0.125	0.131	−0.022
	*p*-value	0.128	0.160	0.140	0.806
Depression scores (points)	Coeff.	0.470**	0.389*	0.485**	0.121
	*p*-value	**< 0.001**	**< 0.001**	**< 0.001**	0.174
Physical activity (METS)	Coeff.	0.057	0.078	0.051	−0.091
	*p*-value	0.522	0.382	0.569	0.307
Smoking (No. of cigarettes/month)	Coeff.	0.079	0.039	0.145	−0.012
	*p*-value	0.375	0.665	0.102	0.895
Waist circumference (cm)	Coeff.	−0.083	−0.063	−0.123	0.203^*^
	*p*-value	0.352	0.480	0.167	**0.022**
Total body fat (%)	Coeff.	−0.062	−0.067	−0.023	−0.096
	*p*-value	0.489	0.449	0.795	0.283

n = 128 Subjects; Coeff.: Spearman’s Rho coefficient; mg/dl: Milligrams per deciliter; Cm: Centimeters; %: Percentage; No. Number; Sig.: * Significance < 0.05 level (Two-sided); ** Significance < 0.01 level (Two-sided).

Subjects taking psychotropic drugs showed higher levels of hyperactivity and impulsivity, both individually and together. Serum UA levels were higher in men and those with metabolic syndrome (see Table 4).

**Table 4:** Hyperactivity, impulsivity symptoms, and serum uric acid, stratified by categorical covariates.

		*Hyperactivity/impulsivity* *(points)*				*Hyperactivity* *(points)*			*Impulsivity* *(points)*				*Serum uric acid* *(mg/dl)*	
Variables	*n*	Median	IQR	P*		Median	IQR	P*	Median	IQR	P*	Mean	(S.E.)	P*
*Biological sex*														
Male	43	15.0	12.0, 25.0			12.0	9.0, 16.0		4.0	3.0, 8.0		5.5	(1.5)	
Female	85	19.0	12.0, 25.5	0.400		13.0	9.5, 18.0	0.378	6.0	3.0, 8.0	0.158	4.6	(1.1)	**< 0.01**
*Use of psychotropic*														
Absence	86	16.0	10.8, 22.3			10.0	8.0, 15.0		5.0	2.0, 8.0		4.9	(1.4)	
Presence	42	22.5	17.8, 27.0	**< 0.01**		15.0	13.0, 9.0	**< 0.01**	7.0	4.0, 10.0	**0.017**	5.1	(1.3)	0.614
*Diagnosis of obesity*														
Absence	28	18.0	11.0, 26.5			13.5	8.0, 18.8		6.5	3.0, 8.8		4.7	1.3	
Presence	100	18.0	12.0, 24.8	0.869		13.0	9.3, 16.8	0.883	6.0	3.0, 8.0	0.779	5.0	1.4	0.207
*Diagnosis of Metabolic syndrome*														
Absence	99	18.0	12.0, 25.0			13.0	9.0, 18.0		6.0	2.0, 8.0		4.8	(1.2)	
Presence	29	18.0	11.5, 26.5	0.811	11.0	8.5, 18.0	0.833	6.0		3.5, 8.0	0.850	5.5	(1.6)	**< 0.01**

* Two-sided significance. IQR: Interquartile Range: Quartile 25, quartile 75.

The positive association between serum UA levels and hyperactivity/impulsivity symptoms and hyperactivity symptoms remained significant after adjustment for potential confounding variables. Thus, for every one mg/dl increase in serum UA levels, there was a 1.5-point increase in hyperactivity/impulsivity symptoms and a 1-point increase in hyperactivity symptoms. Multivariate models explain 37.1% and 28.3% of the variability found in the hyperactivity/impulsivity and hyperactivity symptoms, respectively, as shown in Tables 5 and 6.

**Table 5:** Multivariate general linear model for hyperactivity/impulsivity symptoms.

Variables	Coefficients β	*p*	95% confidence interval	
Constant	8.322	0.136	−2.643	19.283
Serum uric acid (mg/dl)	1.510	**0.002**	0.549	2.470
Dietary protein (g/1000 kcal)	−0.033	0.362	−0.104	0.038
Dietary fructose (g/1000 kcal)	−0.143	0.332	−0.434	0.148
Added sugar (g/1000 kcal)	0.365	0.146	−0.129	0.860
Dietary total fiber (g/1000 kcal)	−0.093	0.466	−0.346	0.159
Dietary Vitamin C (mg/1000 kcal)	0.008	0.216	−0.005	0.021
Dietary Zinc (mg/1000 kcal)	0.014	0.119	−0.004	0.031
Waist circumference (cm)	−0.064	0.215	−0.165	0.037
Depression score (points)	0.307	**< 0.000**	0.211	0.402
Age (years)	0.160	**0.046**	0.003	0.317
Use of psychotropic drugs	4.246	**0.001**	1.685	6.806
Female sex*	−1.496	0.269	−1.174	4.167
Adjusted R^2^	0.371			
Model p-value		**< 0.001**		

*Compared with male sex, g/1000 kcal: Grams per 1000 kilocalories consumed.

**Table 6:** Multivariate general linear model for hyperactivity symptoms.

Variables	Coefficients β	*p*	95% confidence interval	
Constant	8.806	0.023	1.264	16.347
Serum uric acid (mg/dl)	1.007	**0.003**	0.347	1.668
Dietary protein (g/1000 kcal)	−0.032	0.199	−0.081	0.017
Dietary fructose (g/1000 kcal)	−0.059	0.562	−0.259	0.141
Added sugar (g/1000 kcal)	0.255	0.141	−0.085	0.595
Dietary total fiber (g/1000 kcal)	−0.069	0.430	−0.243	0.104
Dietary Vitamin C (mg/1000 kcal)	0.005	0.246	−0.004	0.014
Dietary Zinc (mg/1000 kcal)	0.008	0.201	−0.004	0.020
Waist circumference (cm)	−0.053	0.136	−0.122	0.017
Depression score (points)	0.151	**< 0.000**	0.085	0.217
Age (years)	0.092	0.095	−0.016	0.200
Use of psychotropic drugs	2.848	**0.002**	1.087	4.610
Female sex*	−1.080	0.247	−0.757	2.917
Adjusted R^2^	0.283			
Model p-value		**< 0.001**		

*Compared with male sex, g/1000 kcal: Grams per 1000 kilocalories consumed.

## 4. Discussion

The present findings are significant, revealing that as serum UA levels increase, so does hyperactivity and impulsivity. Despite the multivariate model's modest prediction of one-third of hyperactivity/impulsivity, these associations remained statistically significant after adjustment for known confounders, providing valuable insights.

A mixed strategy was employed for selecting covariates in the multivariate models. Initially, a bivariate association of p ≤ 0.20 was considered, as suggested by Bursac et al. [43], to avoid prematurely excluding potentially relevant predictors. However, final variable inclusion in the multivariate models was primarily guided by clinical relevance, epidemiological consistency, and theoretical support, according to Gregorich et al. [44], who argue that covariate exclusion should not rely solely on collinearity or statistical significance, but instead on theoretical and scientific coherence. General linear models were run because multivariate linear regression was not suitable. For instance, serum uric acid was retained not only due to its bivariate significance but also based on its established physiological link to hypothalamic and hippocampal inflammation, increased impulsivity, disinhibition, excitement seeking and behavioral disorders in experimental models, but also related with low attention span, hyperactivity, impulsivity, and problems with anger control found in children and adults with ADHD [45,46].

Given the multifactorial nature of mental disorders, it is expected that multivariate models in observational studies will account for only a limited proportion of the clinical phenomenon’s variance. The Hierarchical Taxonomy of Psychopathology (HiTOP) model supports the notion that psychopathological symptoms covariate, grouping and forming spectra to hierarchically structure them into dimensions, such as internalizing and externalizing, rather than being strictly defined by categorical diagnostic structures. This perspective also implies that explained variance by models based on categorical clinical variables may exhibit moderate R² values without compromising their clinical utility or theoretical validity [47].

To our knowledge, this is the first study to investigate the relationship between serum UA concentrations and hyperactivity and impulsivity, examining both factors together and separately. The use of a multivariate approach that accounts for dietary factors, unhealthy lifestyle behaviors, and disease indicators enhances measurement precision, making a significant contribution to the field.

The positive correlation between serum UA and hyperactivity was consistent with two reports by Barrera et al. in preschool children [6] and developing rats [48]. However, the present study is the first to adjust for a wide range of known confounders. Although Barrera et al. also found a positive correlation between serum UA concentration and impulsivity in children, Bartoli et al. did not see it in adults with psychopathologies, a sample similar to the present study [22].

Impulsivity can be assessed as a trait or behavior, with behavioral impulsivity further divided into reflective, motor, and decision-making types [49]. The ASRS evaluates motor impulsivity, while other scales evaluate decision-making impulsivity [50] or trait impulsivity [5]. This likely explains the lack of correlation in the present study compared with Barrera´s study. The Adult ADHD Self-Report Scale (ASRS) was initially developed as a screening tool for ADHD, rather than a diagnostic instrument *per se*. However, other studies have validated and demonstrated that the ASRS performs robustly as a dimensional measure of hyperactivity and impulsivity symptoms, particularly when used as a continuous scale in psychiatric and general population samples.

Kessler et al. found an overall concordance by total classification accuracy of 97.9%, a Cohen’s κ of 0.76, and an AUC of 0.84 after comparing ASRS with DSM-IV criteria [51]. Then, Adler et al. demonstrated high internal consistency between the ADHD Rating Scale and ASRS, for both patient- and investigator-rated versions, with Cronbach’s alpha coefficients of 0.88 and 0.89, respectively. High intra-class correlation coefficient between scales for total scores (0.84), and agreement for individual items with kappa coefficients for all items (p < 0.001) [29]. Furthermore, the ASRS has demonstrated dimensional sensitivity to ADHD-like symptomatology even in populations with primary psychiatric conditions such as major depressive disorder, supporting its use for monitoring symptom severity in comorbid contexts [52].

Moreover, our use of the ASRS is grounded in dimensional models of psychopathology such as the Hierarchical Taxonomy of Psychopathology (HiTOP) [4,47] and the Research Domain Criteria (RDoC) framework [53], both of which emphasize the importance of assessing core symptoms across diagnostic boundaries. From this perspective, impulsivity and hyperactivity are not exclusively tied to categorical ADHD diagnoses but rather represent transdiagnostic dimensions relevant to multiple psychiatric syndromes. Using symptom-level dimensional assessments enables us to minimize diagnostic heterogeneity and more accurately capture underlying psychopathological mechanisms.

As reviewed by Creswell et al., traditional self-report measures of impulsivity, such as the Barratt Impulsiveness Scale-11, primarily assess trait-like dimensions of impulsivity, which may not accurately reflect the clinical state or change over time. This contrasts with tools like the ASRS, which capture current symptomatology and may be better suited for psychiatric samples with varying clinical states [54]. Additionally, it is self-applied to assess hyperactivity and impulsivity symptoms at a low cost, is less time-consuming, and thus a feasible evaluation [29].

Adults with psychopathology in the present sample had a mean value of serum UA of 4.9 ± 1.3 mg/dl, below the hyperuricemia cut-off (> 6.9 mg/dl) [28] but higher compared to subjects without medical conditions, medication, or psychoactive substances which affect the UA metabolism or alcohol consumption (3.51 ± 0.97) [55]. The above result might be because subjects with bipolar disorder have been reported to have high serum UA levels (5.43 ± 2.06 mg/dl) [55]. In contrast, low serum UA levels were reported in subjects with current major depressive disorder and anxiety disorder [56]. The patients included in the current study may present both externalizing and internalizing spectrum disorders [4], which could influence the mean.

Subjects who used psychotropic drugs scored higher symptoms of hyperactivity/impulsivity together and separated, consistent with the report of the use of antidepressants associated with behavioral activation: hyperactivity, agitation, aggression, and suicidal ideation [57], while the serum UA levels were higher in men than women and in those with metabolic syndrome as expected [28].

Regarding the dietary intake of nutrients and UA, the total protein, carbohydrate, and fat intake in the present study did not correlate with impulsivity/hyperactivity symptoms or UA, consistent with no difference among macronutrients between groups in a study from Salvat et al., with children diagnosed with ADHD with hyperactivity symptoms compared with their matched-controls [58]. The protein is a precursor of serum UA; thus, it was expected that the protein would correlate with UA. However, these results were consistent with a lack of association between hyperuricemia and total protein intake in Chinese adults from a community reported by Villegas et al. [33]. Villegas found a positive association between hyperuricemia and animal protein intake; however, our study employed the 24-hour recall method, which does not differentiate between vegetal or animal protein sources. This analysis also requires a food frequency questionnaire, which was not applied.

In the present study, dietary fructose negatively correlated with serum UA levels. This contradicts several reports that have confirmed excessive fructose intake can increase fasting serum UA levels [45,59], especially if it comes from corn syrup high in fructose and added sugar [15,28]. However, the added sugar intake from our patients was low (median of 2.4% from energy; IQR 1.5, 3.6), below the < 6% from energy intake recommended by Dietary Guidelines for Americans 2020–2025 [60] and also according to the Dietary Guidelines for the Mexican population which recommends avoiding added sugars [61]. Moreover, fructose is also contained in fruits along with other dietary factors, such as vitamin C, which help to block oxidative stress and xanthine oxidase activation from UA derived from fructose. Additionally, vitamin C and polyphenols can also reduce renal reabsorption and increase UA excretion. Fiber can delay fructose digestion, reducing its absorption velocity in the small intestine [15,62]. The subjects evaluated had an adequate intake of dietary fiber, with a median of 12 g/1000 kcal (IQR of 9.4 g/1000 kcal, 16.6 g/1000 kcal), which is close to the recommended intake of 14 g/1000 kcal [63]. Those possible mechanisms were confirmed by a positive correlation between dietary fructose and vitamin C, as well as with total fiber intake (Rho = 0.271, p = 0.002 and Rho = 0.344, p < 0.001, respectively). Interestingly, dietary fiber also reduces UA by interfering with the absorption of purines, increasing bowel movements, and promoting the excretion of UA [64], which may have occurred in the present study, as serum UA negatively correlated with dietary vitamin C and total fiber.

The UA is primarily produced in the liver and is found peripherally in the blood vessels, allowing it to pass freely to the brain [65]. In experimental models, fructose reduced the activation (phosphorylation) of the insulin receptor (IR-A) and insulin receptor substrate-2 (IRS-2), leading to insulin resistance in the brain [66]. Fructose also causes oxidative stress, impairs mitochondrial function, and contributes to inflammation, potentially reducing oxidative phosphorylation and energy production, which leads to a shift toward glycolysis [67,68]. This shift is particularly notable in the brain, where glycolysis is limited [69]. This low energy production in the brain resulted in decreased prefrontal cortical activity and increased impulsivity in animals [70] and in humans [71].

The present data showed that serum UA was negatively associated with copper, consistent with a report that copper and zinc reduce UA [15]. Although dietary zinc followed the expected negative relationship with UA [72], it did not reach statistical significance, possibly due to the limited sample size. Notably, dietary zinc showed a statistically significant negative correlation with the hyperactivity score but a borderline negative correlation with the combined hyperactivity/impulsivity score. A negative association between zinc deficiency and ADHD, particularly regarding hyperactivity and impulsivity symptoms, has been documented in both animal and human studies. This relationship has generated interest in the potential for dietary zinc intake to prevent or co-treat ADHD [73–75].

Zinc possesses strong antioxidant and anti-inflammatory properties, functions as a cofactor for metalloenzymes and neurogenesis, and activates receptors such as NMDA (N-methyl-d-aspartate), GABAA (γ-aminobutyric acid type A), AMPA (α-amino-3-hydroxy-5-methyl-4-isoxazole propionic acid), and glycine. These actions influence synaptic plasticity and transmission, and zinc plays a significant role in the dopamine pathway [76,77].

The dopamine transporter has a high affinity for the zinc-binding site on its extracellular surface, leading to potent inhibition of dopamine reuptake. Zinc also regulates dopamine and melatonin metabolism via the dopamine transporter. Reduced striatal zinc, due to insufficient dietary intake, diminishes dopamine transporter binding and alters cocaine effects [76,78,79]. Consequently, lower zinc levels may contribute to increased inattentiveness, hyperactivity, restless leg syndrome, and other sleep disturbances. Zinc supplementation may also modulate pyridoxal phosphate, derived from dietary pyridoxine, which is involved in the conversion of tryptophan to serotonin. This process can enhance serotonergic function and reduce impulsivity. Patient response to zinc supplementation is influenced by baseline zinc levels, body mass index, age, and symptom severity. Notably, both zinc deficiency and excess can disrupt dopaminergic metabolism and negatively impact mental health, demonstrating a U-shaped effect [79].

Although one-third of UA is produced from food-containing purines [80,81], the association between dietary components and UA concentration is possibly more complex than estimating the purine intake; for example, beer [28] and alcohol intake increase UA, but caffeine decreases UA levels in the organism independently of their purine content [13,15]. Caffeine is a psychostimulant that elicits non-selective adenosine receptor antagonism of A1 and A2A receptors expressed in the brain [82]. In fact, adenosine receptor knockout mice have demonstrated that the adenosine receptor A2A regulates motor activity, confirming that caffeine and its xanthines increase locomotion and, consequently, hyperactivity [83]. However, caffeine consumption did not correlate with UA or outcomes as expected in present study. These findings may be attributed to the low caffeine consumption (median of 1.3 mg/day in 52% of the subjects). On the other hand, the expected positive association between alcohol consumption and UA (due to the ATP degradation to AMP) [13] was also not observed. Conversely, impulsivity and alcoholism were positively associated in the present study, as expected [84]. Alcohol decreases dopamine transporter binding and disturbs dopamine synaptic transport [85].

The age of patients did not correlate with hyperactivity and impulsivity, either together or separately, possibly due to the low age range of our inclusion criteria (18–44 years), which was selected based on the age range of the ASRS scale validation. However, in the multivariate models, age was a significant predictor.

The positive and significant correlation between depression and impulsivity, and between depression and hyperactivity in the present study, was also consistent with ADHD symptoms and depression in subjects from the community with a similar age range [86]. This association can be explained because impulsivity and anhedonia (a key symptom of depression) share common neural substrates, which include both low tonic mesolimbic dopamine activity and a reduced phasic mesolimbic dopamine response to motivation during anticipation of reward and associative learning [87].

Moderate to vigorous intensity of physical activity has been inversely related to UA levels [19], but no association was found, likely because only 46% of the sample engaged in physical activity. In individuals with psychopathology, Bartoli et al. reported that obesity measured by body mass index (BMI) positively correlated with UA levels [22], but we did not find it. However, central obesity, measured by waist circumference, was positively correlated with UA levels, as previously reported [39]. Smoking has been positively associated with serum UA levels [21], but in the present study, there was no association; although smoking was measured by self-report, this has been reported to be reliable [88].

The independent association of UA and hyperactivity/impulsivity score, or hyperactivity score, could be explained by the alteration in the purinergic system, where the ATP generated in the mitochondria would be expected to form AMP and, in turn, adenosine, which activates A2AR receptors and could reduce the affinity and signal transduction of D2R, resulting in reduced motor activity [89]. However, in the brain, a diversion from AMP towards IMP occurs instead of adenosine, like what happens in the liver to obtain UA [89]. Interestingly, when UA was formed instead of adenosine, it increased locomotor activity, exploratory activity, and impulsivity in rats [5,89,90]. These provide biological plausibility for the association of high serum UA with pathological gambling [50], substance use disorder [91], and bipolar disorder [92]. Moreover, high serum UA was reported in children [6] and adults [93] with hyperactivity, impulsivity, low attention span, poor anger control, and sleep disorders [94], all related to ADHD. Consistent with the above mechanisms, patients with ADHD exhibited reduced D2 receptors in the left ventral striatum (involved in behavior), the left side of the brain (a region of the dopamine reward pathways), and the left hypothalamus (involved in memory) compared with healthy adults [95,96]. This lower availability of D2 receptors also causes decreased prefrontal cortical activity and decreased ability to inhibit behaviors, resulting in increased impulsivity in animal models [97]. Several clinical trials have reported that allopurinol, used as a co-treatment in patients with bipolar disorder, reduced UA blood concentration and manic symptoms [98].

### 4.1. Study limitations

Impulsivity may be related to emotional dysregulation; however, this relationship was not adjusted for [99]. The ASRS scale has a high specificity of 99.5% but its sensitivity is only 68.7%. This scale may be subject to recall bias [51]. The use of the 24-h dietary recall is another scale that may be subject to recall bias, which may also under- or over-report recent dietary intake. In the present research, a multiple-pass method was applied, which includes food consumed by the respondent on the day before the interview and a forgotten food list, among other details, to reduce bias [31].

The omega-3 fatty acids regulate factors of neurotransmission and downregulate neuroinflammation, reduce oxidative stress, and promote neural survival. However, the omega-3 very long-chain polyunsaturated fatty acids, docosahexaenoic acid (DHA) and eicosapentaenoic acid (EPA), have more potent effects compared with their precursor, alpha-linolenic acid [32,100]. Nevertheless, the entire Omega-3 family of fatty acids was estimated, which may have led to an overestimation of their intake. The food processor software did not report purine content because its USDA National Nutrient Database does not include this content [101]. In addition, this study did not address artificial sweeteners, colors, flavors, preservatives, additives, or dietary interventions [102,103], which are desirable topics for future studies on psychiatric conditions.

### 4.2. Study Strengths

The evaluation of the relationship among serum UA levels, hyperactivity, and impulsivity was adjusted by quantitative measurement of potential confounders. Additionally, variable precision was improved. For example, obesity was previously determined by BMI and correlated with serum UA levels in adults with psychopathologies [22], but the present study reported a more precise measurement (total body fat in percentage). An individual with a normal BMI may be misclassified as “normal” but may have excessive total body fat. Conversely, a subject with high muscle mass may be diagnosed as having obesity [104].

Regarding the nutrient intake that may reduce or increase serum UA, it was estimated with a specialized software, excluding subjects who were taking dietary supplements. Likewise, only subjects with normal hepatic and renal function tests were selected [26].

Due to the cross-sectional design of this study, causality cannot be established. In addition, dietary patterns and psychiatric symptoms can influence one another in both directions. Although the present results should be interpreted with caution, the 24-hour recall was used because the aim was to evaluate the relationship between recent dietary intake and serum uric acid concentrations with the clinical assessment of current hyperactivity and impulsivity symptoms. An experimental study in young adults (25 ± 1 years) showed that a nucleotide-rich mixed meal resulted in a higher relative rise of serum uric acid concentrations (16 ± 4% versus 9 ± 2%, p > 0.05), displaying more persistent elevations after 24h in females compared with males, not reaching the baseline levels after 24h as it was observed in males (female levels of serum uric acid concentrations at baseline: 264 ± 14 µmol/L vs. after 24h: 292 ± 21 µmol/L) [105]. Moreover, in the present study, more than half of our sample were female patients. Further studies aimed at evaluating the relationship between hyperactivity and impulsivity symptoms and serum uric acid concentrations should consider habitual dietary intake using food frequency questionnaires, ideally with portion sizes, or dietary biomarkers, while adjusting for sex and other confounders, to elucidate this link.

According to present results, it might be advisable to moderate alcohol consumption, as this increases depression and impulsivity. Additionally, adults with hyperactivity symptoms should also reduce their intake of fructose, which is more concentrated in processed foods, added sugar, and fruit juice [15,62]. Conversely, increasing the consumption of natural, less mature fruit is associated with lower fructose content and other factors that mitigate the deleterious effects of fructose, as they also contain fiber, vitamin C, and minerals [15,62]. Moreover, high amounts of fruit often reduce refined and added sugar intake in humans, so the final amount of fructose intake may be low [62]. In addition, increasing dietary fiber, vitamin C, copper, and zinc intake appears to be beneficial for the general health.

The serum UA could be a helpful marker of the clinical state for the hyperactivity/impulsivity symptoms in psychopathological disorders [46,106,107], and this study gives more substantial evidence of its possible role. These results may have only internal generalizability. Future investigations should determine its prediction in specific psychiatric comorbidities such as bipolar disorder, major depression, generalized anxiety disorder, ADHD, and borderline personality disorder, among others.

## 5. Conclusions

The results showed that serum UA is associated with symptoms of hyperactivity/impulsivity, both when scored together and when scored separately as hyperactivity. After considering nutrient and medication intake, depression, waist circumference, biological sex, and age as confounders, these associations remained significant. Fructose, vitamin C, fiber, copper, and zinc may play a role in modulating serum UA and symptoms of hyperactivity/impulsivity; however, this should be established.
